# Gangliosides and Neuroblastomas

**DOI:** 10.3390/ijms21155313

**Published:** 2020-07-27

**Authors:** Cara-Lynne Schengrund

**Affiliations:** Department of Biochemistry and Molecular Biology, College of Medicine, Pennsylvania State University, Hershey, PA 17033, USA; cxs8@psu.edu

**Keywords:** ganglioside, neuroblastoma, glycosyltransferases, glycosidases

## Abstract

The focus of this review is the ganglio-series of glycosphingolipids found in neuroblastoma (NB) and the myriad of unanswered questions associated with their possible role(s) in this cancer. NB is one of the more common solid malignancies of children. Five-year survival for those diagnosed with low risk NB is 90–95%, while that for children with high-risk NB is around 40–50%. Much of the survival rate reflects age of diagnosis with children under a year having a much better prognosis than those over two. Identification of expression of GD2 on the surface of most NB cells led to studies of the effectiveness and subsequent approval of anti-GD2 antibodies as a treatment modality. Despite much success, a subset of patients, possibly those whose tumors fail to express concentrations of gangliosides such as GD1b and GT1b found in tumors from patients with a good prognosis, have tumors refractory to treatment. These observations support discussion of what is known about control of ganglioside synthesis, and their actual functions in NB, as well as their possible relationship to treatment response.

## 1. Introduction to Neuroblastoma and the Biological Importance of Gangliosides

While hundreds of glycosphingolipids have been identified [1], this review will focus on the ganglio-series of glycosphingolipids (GSLs) found in neuroblastoma (NB) and the myriad of unanswered questions still associated with their possible role(s) in this cancer. NB is of interest because it is one of the more common solid malignancies of children accounting for ~7% of all pediatric cancers (International Neuroblastoma Risk Group). For a description of the development of NBs from neural crest cells and the heterogeneity of the cells that comprise the tumors see Kholodenko et al. [2]. Two different systems have been developed for staging NBs: The International Neuroblastoma Staging System (INSS) [3] classifies NBs based on what is found at the time of surgery while the International Neuroblastoma Risk Group Staging System (INRGSS) [4] uses imaging, exams, and biopsies. In both staging systems those whose tumors have metastasized to distant parts of the body have the poorest prognosis unless the child is less than 18 months of age (Table 1). While the five-year survival rate for those diagnosed with low-risk NB is 90–95%, that for children with high-risk NB is around 40–50% [5]. Interestingly, much of the survival rate reflects the age of diagnosis with children less than a year having a much better prognosis than those over the age of two [6].

**Figure 1:**
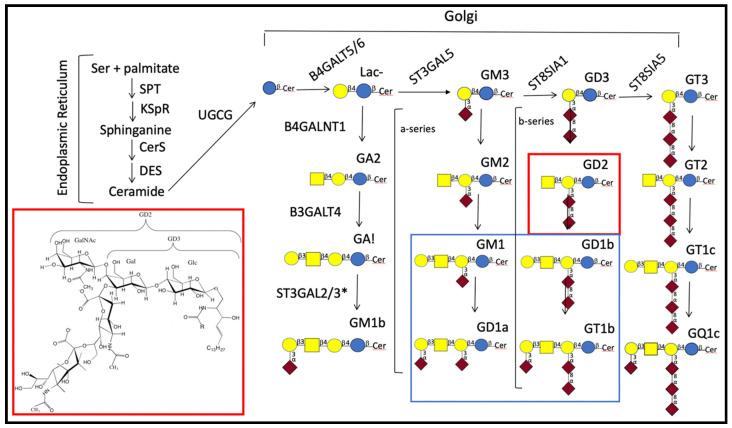
Synthetic pathways for common central nervous system (CNS) gangliosides. Major CNS gangliosides are enclosed by the rectangle outlined in blue and the structure and place of GD2 in the synthesis of gangliosides by red; 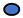
—glucose; 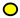
—galactose; 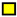
—N-acetylgalactosamine; and 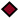
—sialic acid [11]. GA1, GA2, and GA3 indicate asialylated gangliosides. Nomenclature used for ganglioside series gangliosides was developed by Svennerholm [12]. Brackets indicate gangliosides that are in the a-series (one sialic residue linked ±2–3 to the galactose linked β1–4 to glucose) or in the b-series (two sialosyl residues linked to the galactose as shown). Gene abbreviations are those of the Human Genome Organization (HUGO) gene nomenclature committee (https://www.genenames.org/tools/multi-symbol-checker/). Enzymes indicated are: B4GALT5/B4GALT6 [13,14], UDP-galactose: glucosyceramide β1–4 galactosyl transferase (lactosylceramide synthase); B4GALNT1, UDP-GalNAc:LacCer/GM3/GD3/GT3 β1–4 N-acetylgalactosaminyl transferase (ganglioside GA2, GM2, GD2, synthase); B3GALT4, UDP-galactose:GA2/GM2/GD2/GT2 β1–3 galactosyl transferase (ganglioside GA1, GM1a, GD1b, and GT1c synthase); CerS, ceramide synthase; DES, dihydroceramide desaturase; UGCG, UDP-glucose:ceramide β1-1′-glucosyl transferase; KSpR, 3-ketosphinganine reductase; SPT, serine-palmitoyl transferase; ST3GAL5, CMP-sialic acid:lactosylceramide α2–3 sialyltransferase (GM3 synthase); ST8SIA1, CMP-sialic acid:GM3 α2–8-sialyltransferase (GD3 synthase); ST8SIA5, CMP-sialic acid:GD3 α2–8-sialyltransferase (GT3 synthase). * ST3GAL2/3 are needed in mice for synthesis of D1a and T1b [15], but the specificity of these enzymes in humans is still under study [16]. For a discussion of similarities and differences in genes needed for ganglioside synthesis in mice and humans see Schnaar [17]. For characterization of GM1b see [18].

Gangliosides, sialylated glycosphingolipids (GSLs, see Figure 1), are found in the greatest concentration in the central nervous system (CNS) where their need for normal neural function/development was initially proven in studies of mutant mice. While mice unable to express functional ST8SIA1 and B4GALNT1 appeared grossly normal at birth even though they could synthesize just GM3 (Figure 1), they were found to have shortened life spans and to experience lethal audiogenic seizures upon exposure to a sudden noise [7]. Interestingly, disruption of either gene alone, had markedly less effect on the mice with *St8sia1^-/-^* mice appearing to develop normally while *B4galnt1^-/-^* mice had defects in myelination and little response, non-lethal, to sound stimulus [7]. The question of whether gangliosides were essential for humans or whether other glycoconjugates might function in their place was initially answered with identification of an infantile-onset symptomatic epilepsy syndrome caused by a homozygous loss-of-function mutation in the gene encoding GM3 synthase (*ST3GAL5*) needed for synthesis of the more complex sialylated CNS gangliosides [8]. While at birth, children with a defect in this gene appear normal, the majority fail a newborn hearing test. Additional symptoms included somatic growth failure, progressive microcephaly, global developmental delay, involuntary movements, irritability, and a reduction in median life span to 23.5 years [9]. Humans lacking functional B4GALNT1 appear normal at birth but tend to have some intellectual impairment and to undergo a gradual loss of lower limb function as they move into middle age [10].

In contrast to gangliosides expressed in mature neurons, NBs often express simplified ganglioside patterns lacking or having reduced concentrations of the more complex b-series gangliosides GD1b and GT1b [19,20]. b-Series gangliosides are defined by the presence of a sialic acid disaccharide linked ±2–3 to the galactose linked β1–4 to glucosylceramide (glc-cer) while a-series have just one as shown in Figure 1. An important historical milestone in the study of NB was identification of the expression of less complex gangliosides such as GD2 on the surface of most NB cells [21,22,23]. Over time, confirmation of this observation, coupled with the low expression of GD2 in healthy tissue led to studies of the effectiveness and subsequent approval of the use of anti-GD2 antibodies to treat patients with tumors expressing GD2 (for a recent review see [24]). This brief introduction supports interrogation of the underlying role(s) of gangliosides in NB. For example: (1) Do gangliosides on neuroblastoma cells reflect their stage of differentiation or type of neuron they might become provided the appropriate environment? (2) Why do NB cells generally have elevated levels of GD2? Does it have a particular function?; and (3) Do indicators used in the prognosis of NB provide insights into why (a) different tumors respond differently to treatment and (b) younger children with NB tend to have a better prognosis?

## 2. Ganglioside Structure and Biosynthesis

Gangliosides are comprised of a lipid tail, consisting of sphingosine and a fatty acid, and a polar headgroup comprised of sialylated ganglio-oligosaccharides (Figure 1). While differentiation of neurons is accompanied by defined changes in their oligosaccharide composition synthesized by the action of specific glycosyl transferases (Figure 1), it is important to understand how controls on those changes can be affected by transport proteins needed to move the GSL being synthesized from one site to the location of the enzyme catalyzing the next step. For a recent review of steps involved in synthesis of ceramide, the lipid carrier of the oligosaccharide moiety of GSLs, see Ogretmen [25]. Ceramide produced can be used for the synthesis of sphingomyelin or GSLs. Conversion to glucosyl glc-cer requires it to associate with the endoplasmic surface of the Golgi, a non-vesicular process [26], thought to be facilitated by the ceramide transport protein (CERT) [27,28] that recognizes D-erythro-C16-ceramide with C_14_-C_20_ amide-acyl chains [29]. After glucosylation in a reaction catalyzed by UGCG (UDP-glucose:ceramide β1-1′-glucosyl transferase), it is transported to the *trans*-Golgi by the glycolipid transfer protein, 4-phosphate adaptor protein 2 (FAPP2) [30]. The structure of FAPP2 is such that it preferentially binds simple glycosphingolipids (GSLs) such as glc- or gal-cer. Ganglioside synthesis from glc-cer depends upon the action of specific glycosyl transferases which rely upon their appropriate localization in the Golgi, and the sphingosine and fatty acid components comprising the ceramide. Evidence for distinct locations is supported by the observation that different glycosyltransferases are found in different complexes in the Golgi [31] which can be affected by levels of their expression [32]. Dependency on the ceramide portion reflects the fact that CNS gangliosides lack the fatty acid diversity found in non-CNS glycolipids, containing predominantly stearic acid [33,34], and either a C18 or C20 sphingosine [34,35]. Table 2 indicates examples of controls for enzymes and transporters needed to catalyze conversion of ceramide to gangliosides found in the mature nervous system.

Once synthesized, gangliosides can be transported to the plasma membrane via the luminal surface of transport vesicles where they can be modified by cell surface glycosyltransferases and glycosidases, or endocytosed [49]. A well-studied plasma membrane [50], lipid raft-associated [51], externally oriented and possibly trans-membrane [52] glycosidase is sialidase, also called neuraminidase. In all there are four types of sialidase (neuraminidase 1–4 (NEU1–4)) each of which catalyzes the hydrolysis of sialosyl residues from different substrates. NEU3 acts on substrates having a hydrophobic aglycone, such as gangliosides, catalyzing hydrolysis of α2–3 linked sialosyl residues found in GM3, GD1a, and GT1b but not those in gangliosides containing a branch point at the adjacent galactose residue such as found in GM1 and GM2. It can also catalyze hydrolysis of the α2–8 linked sialosyl residues found in gangliosides such as GD3, GD1b, and GT1b [53,54]. NEU4 can also catalyze hydrolysis of ganglioside sialic acid residues and is expressed to about the same extent as NEU3 in the brain [55]. However, the intracellular location of the enzymes differs, with NEU4 associated with the endoplasmic reticulum (ER) [56], lysosomes [57], and mitochondria [56,58], as does their expression during development [55]. The result of NEU3 activity is not only a change in ganglioside composition but a change in cell surface charge due to the loss of the negatively charged sialic acid moieties.

In terms of NB, interest in sialidase reflects the fact that early observations indicated that its activity might correlate with oncogenicity of transformed hamster embryo fibroblasts [59] with about 40% of the activity associated with plasma membranes [60]. Subsequently, the plasma membrane isoform of the enzyme was isolated [61], cloned, and characterized [50]. Further study indicated that the isoform named, NEU3 (neuraminidase 3), is expressed by human NB cells where it helps regulate neurite formation [62]. The production of GM1 as a result of NEU3 acting on endogenous gangliosides [63] may account for that effect since GM1 has been shown to affect a number of cellular functions, including neuritogenesis by neuroblastoma cells [64]. However, overexpression of GM1 by pheochromocytoma cells was found to inhibit nerve growth factor signaling due to GM1-induced alterations in membrane fluidity [65]. Another instance of its effect on cell proliferation was seen using mouse fibroblasts (NIH3T3 cells) where its conversion of GM3 to Lac-cer resulted in activation of the epidermal growth factor receptor (EGFR) together with the Src family of protein tyrosine kinases, thereby enhancing the tumorigenicity of the cells [66]. Their results indicated that NEU3 functioned in tumorigenesis via the EGFR/Src signaling pathway and led them to posit that inhibiting NEU3 might be a treatment option for delaying tumor growth by cells expressing GM3 as their major ganglioside. These observations emphasize that care must be taken to note the type of cells studied and their ganglioside components when looking at the effects of NEU3 and of gangliosides in general [67].

## 3. Gangliosides in Neuroblastoma

Observations that prognosis was generally better for patients with tumors expressing higher concentrations of the b-series gangliosides, GD1b and GT1b [19,20], found in nervous tissue of healthy adults [68], provide the basis for discussing how gangliosides appear to affect cell proliferation/differentiation. Support for the early finding of an apparent correlation between level of expression of b-series gangliosides (e.g., GD1b, GT1b) and prognosis was provided by the observation that the hypermethylation of *B3GALT4,* seen in tumor cells from patients classified as stage M and who subsequently died from NB, resulted in its lower gene expression in those patients [48]. Since B3GALT4 catalyzes the first step in the conversion of GD2 to more complex b-series gangliosides it provides an explanation for the previously reported reduction in expression of those gangliosides.

Analyses of the ganglioside composition of developing mouse brains indicated that gangliosides associated with the CNS, primarily GD1b/GT1b and GM1/GD1a (Figure 1), appeared mid-embryogenesis and increased until they reached patterns found in mature brains by about 14 days after birth [69]. Proliferating, neural stem cells express both GD3 as well as measurable levels of GD2 [70]. Interaction of GD3 with the EGF receptor was found to allow neural stem cells to continue to proliferate [71] while studies of breast cancer stem-like cells indicated that activation of the FAK-AKT-ERK-mTOR signaling pathway by GD2 enhanced cell proliferation (Table 3) [72]. These observations support the hypothesis that GD3 and GD2 contribute to the undifferentiated state of neural stem cells (NSCs) and in NB that GD2 contributes to the oncogenic properties. An obvious question raised by these observations is what accounts for the reduction in GD3 synthase found to account for the accumulation of GD2 in human NB cells [73]; also unknown is whether developmental changes seen in ganglioside expression as neural stem cells differentiate occur in cells that give rise to NB tumors that spontaneously regress or indicate a more differentiated clonal cell population. As more is learned about the maturation of neural stem cells and (1) the function of gangliosides involved in their replication and subsequent differentiation, (2) controls on expression and activity of proteins needed for ganglioside synthesis, (3) proteins gangliosides affect, and (4) how each of these is altered in NB cells, the probability of identifying a treatment for NBs and other tumors, expressing tumor-associated gangliosides (e.g., melanomas [73,74] and osteosarcomas [75,76]), refractory to current therapeutics, should improve [2].

In addition to noting the varied effects gangliosides induce via signal transduction, investigators studying neural differentiation are tracing those pathways to identify their effects on transcription (e.g., [90,91,92,97,98]). A more direct effect of a ganglioside on transcription was provided by the observation that GM1 associated with the nuclear lamina of differentiated neurons could bind to acetylated histones on the promoters of *Galnt* and *Neurod1* to influence their activity [99]. Perhaps best understood are the multiple functions of GM1 which can also affect ion transport, functions of G-protein coupled receptors, neuronal differentiation, and the immune system (for a review see [64]). Table 3 depicts some of the functions recently identified for GD2 and other gangliosides found in NB cells and cell behaviors affected.

## 4. Gangliosides in Glycolipid Enriched Microdomains (Lipid Rafts)

The structure of gangliosides allows them to interact with cholesterol and other lipids to give rise to glycolipid enriched microdomains (GEMs), also termed lipid rafts, found in cell membranes [100,101,102,103,104]. The ceramide portion anchors the GSLs into the membrane with their carbohydrate head groups exposed on the surface. Their association with signal transduction proteins/receptors provides them with an ideal location to function in interactions with signaling molecules (e.g., [102,105]) affecting a variety of cell functions (e.g., [106]). It is their apparent ability to modulate signal transduction and hence cell behavior that makes understanding factors affecting their expression essential.

**Figure 2:**
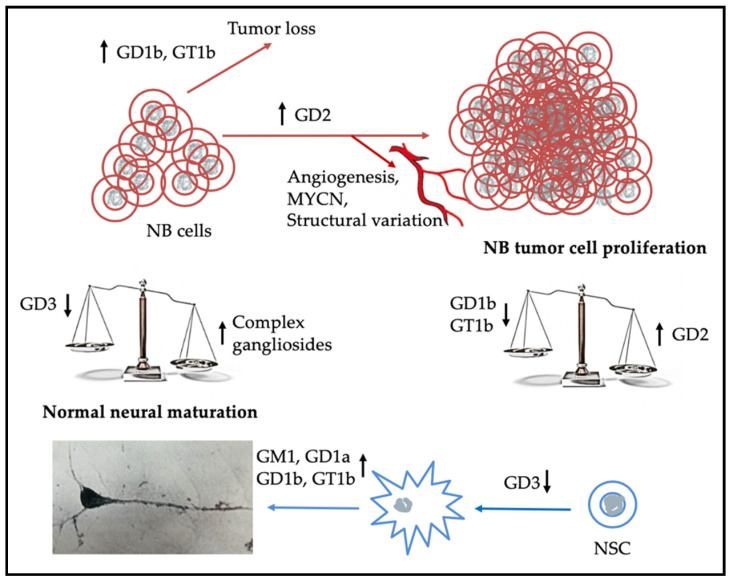
Elevated expression of GD2 and below average expression of b-series gangliosides by NB cells results in cell proliferation giving rise to a tumor that may be refractory to treatment, while children with NBs that express higher concentrations of the more complex b-series gangliosides tend to have a better outcome [19,20]. Unanswered is what is the mechanism for clearance of tumor cells in children whose tumors express more of the b-series gangliosides and regress spontaneously. The lower half of the schematic indicates that as expression of GD3 by neural stem cells (NSCs) decreases and that of the more complex gangliosides increases, they start to differentiate and to express a ganglioside pattern associated with mature neurons [69].

The ability of gangliosides to migrate laterally within the membrane to become components of GEMs, provides the potential for their oligosaccharide moieties to become clustered, thereby providing multiple binding sites for proteins that can recognize/bind multiple carbohydrate moieties. While each carbohydrate–protein interaction may be weak, binding by a single protein with multiple binding sites enhances the strength of the interaction [107]. By the same token, reduction of lateral mobility as a result of overexpression of a ganglioside can inhibit/alter a function. Examples of this possibility are the findings that overexpression of GM1 altered the intracellular localization of nerve growth factor (NGF receptors and membrane fluidity in PC12 cells [65], and in human mammary epithelial cells, it enhanced movement of EGFR from GEMs to caveolae [82]. Evidence that the oligosaccharide portion of a ganglioside can in some instances induce effects analogous to those caused by the intact ganglioside was provided by the observation that the oligosaccharide portion of GM1 alone could induce neurite formation by neuroblastoma cells [86] as well as ameliorate the symptoms of Parkinson’s disease expressed by *B4galnt1*^+/−^ mice [108]. The observations that the oligosaccharide could cross the blood–brain barrier and did not alter total ganglioside concentration supported the conclusion that improvement was due to action of the oligosaccharide with cell proteins. An example of this type of interaction is seen in the recognition and binding of ganglioside glycans by tissue lectins [109]. Due to both the myriad differences that can be induced during synthesis and subsequent modifications of the oligosaccharide portion of gangliosides, identification of their interactions with cell components is imperative for understanding their functions in neuronal maturation and the uncontrolled growth of neuroblastoma cells shown schematically in Figure 2. For example, the finding that b-series gangliosides tend to be enriched in NBs that respond to therapy [19,20] provides a rationale for determining whether their addition or that of GM1, known to enhance neuritogenesis, or the oligosaccharide portions thereof (e.g., [108]), to the tumor would inhibit its growth.

## 5. Gangliosides as Therapeutic Targets in Neuroblastoma

As indicated in the introduction, the presence of enriched amounts of GD2 on the outer surface of the plasma membranes of most NB tumor cells relative to other cells in the body (e.g., [24,89]), supported testing anti-GD2 antibodies as a potential therapeutic for NB [21]. Additional impetus was provided by the observation that the ability of transformed fibroblasts to grow in syngeneic immunocompetent mice was significantly reduced when GM3 and GM2 synthases, needed for synthesis of GD3 and GD2 precursors respectively, were knocked out [110]. While treatment of NB with anti-GD2-antibodies has had success, about one-third of NB patients treated with it do not respond favorably [111]. The observation that some NB cells express low levels of GD2 [112] may account in part for the lack of response. The finding of higher levels of GD2 in the circulation of children with stage 4 NB and those who died of NB, than in those with more differentiated tumors [113], offers another possibility: binding of the anti-GD2 antibody to the circulating GD2 reduces its availability to act on actual tumor cells. Since GD2 is expressed on the majority of NB cells, it has been used and recommended for use to identify NB cells in the circulation and bone marrow as part of a diagnostic test to monitor patient response to NB therapy [114]. The lack of GD2 expression by some NBs, possibly due to their having high GD2 synthase activity and very low levels of GD3 synthase [73], provides a possible explanation for the observation that the presence or absence of GD2 positive cells in the bone marrow and circulation did not correlate with event-free and overall survival [115,116]. That observation, coupled with the finding that GM2/GD2 synthase mRNA could be used as a marker to detect GD2-negative tumor cells present in blood and bone marrow [117] provides an additional and potentially more effective, albeit not included in the INRGSS list [114], marker for identification of treatment response. For a recent review on monitoring response by neuroblastoma patients during immunotherapy see Szanto et al. [118].

The enriched amounts of GD2 present on the outer surface of the plasma membranes of neuroblastoma cells relative to untransformed tissues reduce death of non-neuroblastoma cells when anti-GD2 antibodies are used as a therapeutic treatment. A problem with using the chimeric anti-GD2 antibody Ch14.18 (trade name is Unituxin for that produced in murine myeloma cells and Isquette when produced in Chinese hamster ovary cells) approved for treatment is that its binding by Fc receptors can result in allodynia (relatively opioid-resistant non-neurotropic pain). Cause of the pain was shown to result from Fc-induced complement-dependent cytotoxicity [119]. On the plus side the pain generally clears once treatment is stopped [119]. While use of Fv fragments was found to reduce such unwanted side effects, they bound to GD2 with lower affinity than the intact bivalent antibodies [120]. To develop an anti-GD2-antibody treatment with fewer side effects, site directed PEGylation was used to generate mono-, di-, and tetra-scFv fragments of the U.S. Food and Drug Administration (FDA)-approved 14.18 antibody used to treat NB. While the monomers bound with an affinity comparable to that of the single-chain variable fragments, dimers and tetramers showed a significant increase in affinity for GD2, had increased circulation times, increased penetration of murine tumors, as well as direct cytotoxic effects on GD2-positive tumor cells [121]. These observations support interrogation of the use of multimeric antibody fragments coupled with cytotoxic drugs or radioactive isotopes with effective pharmacokinetic and pharmacodynamic properties for treating tumors resistant to current therapies [121]. Increased cytotoxicity of the multimeric Fv compounds may reflect the fact that multiplicity of binding increased affinity of the PEGylated sc-Fv fragments for their oligosaccharide ligands [107]. Ample proof of the multivalent binding concept is provided by observations of the effectiveness of multivalent oligosaccharide inhibitors at blocking binding by the pentavalent binding subunits of both cholera and Shiga toxin [122,123] to their carbohydrate receptors. The multivalent effect may also answer the question of why tumors with greater levels of GD2 respond more favorably to anti-GD2 immunotherapy [112]—they have more GD2 molecules, clustered in GEMs, available for binding.

Another approach to enhance the efficacy of anti-GD2 antibodies is to increase GD2 expression by NB cells, thereby increasing the probability that the antibody will (1) adhere to the cell and (2) bind with greater affinity if spacing permits it to adhere to more than one GD2 molecule. The histone deacetylase (HDAC) inhibitor Vorinostat was found to work synergistically with anti-GD2 antibodies in the treatment of neuroblastoma [124]. While the HDAC inhibitor enhanced expression of GD2 it did not induce significant changes in GD2 synthase mRNA but did increase GD2 synthase protein levels. Subsequently, it was found that when NB cells were grown in media supplemented with both N-acetylneuraminic acid and Vorinostat, expression of GD2 was enhanced [125]. Comparable enhancement was not seen when the cells were grown in the presence of added sialic acid or Vorinostat alone. Interestingly, the HDAC inhibitor upregulated expression of ST3GAL5 and ST8SIA1, the sialyltransferases that generate GM3 and GD3 gangliosides, substrates for GD2 synthase. Together these observations indicate that the use of sialic acid analogues and HDAC inhibitors to enhance GD2 expression followed by anti-GD2 targeted immunotherapy might be effective for patients with high-risk tumors that are refractory to treatment [125]. They also indicate that post-translational modification must be considered when looking at possible therapies. Regardless of the method used, an effective way to identify cells that survive the initial round of anti-GD2 antibody treatment is needed as residual cells can subsequently multiply and cause patient relapse.

## 6. Examples of Additional Prognostic Markers and How They May Act Synergistically with Gangliosides in NB

The INRGSS uses multiple analyses to arrive at a diagnosis for a NB patient [4]. Two such indicators that have long been used are tumor histology, the more “normal” the cells look, the better the prognosis, and DNA ploidy, hyperdiploid cells are associated with a better prognosis for children less than two [114]. Newer analyses include genetic analyses. Investigation of possible genetic causes of familial NB indicated that mutations in *ALK* (anaplastic lymphoma kinase) was the predominant causal agent [126]. To confirm this conclusion, they showed that when expression of *ALK* mRNA by NB cells was knocked out, it resulted in inhibition of growth by cells having either mutant or amplified *ALK*, as well as those with wild-type. ALK upregulates MYCN transcription [127] and the mechanism for this is being studied with a view to developing inhibitors [128].

*MYCN* (V-myc myelocytomatosis viral-related oncogene, neuroblastoma derived) amplification, associated with spontaneous NB and found in about 20% of all NBs, is associated with a poor prognosis [4,129,130]; although children with tumors without MYCN amplification can also do poorly [131,132]. While the prognostic value of MYCN is highly context-dependent [133], its determination has been recommended as a mandatory step when determining treatment [4,134]. Overexpression of MYCN allows it to form heterodimers with MYC-associated protein X (MAX) which permits it to act as a transcriptional factor and support continued tumor growth [135]. The effect of MYCN-MAX on tumor growth coupled with the observation that GD2 is able to enhance angiogenesis [93], thereby increasing the supply of blood borne nutrients needed by proliferating tumor cells, supports the hypothesis that they act synergistically to enhance/maintain cell growth. The finding that children expressing both amplified *MYCN* oncogene and kinase mutations in *ALK* were reported to have <15% survival rate after three years underscores the need to develop effective therapeutic approaches for inhibiting both.

A number of genetic abnormalities (e.g., gain of chromosome 17q, loss of 1p and 11q) have been identified and used to help predict clinical outcome (e.g., [136,137]). Although chromosome deletions in 1p and 11q are associated with a less favorable prognosis while the entire loss of chromosome 11q is linked to a good income, genetic abnormalities have not always been effective at predicting prognosis. Finding that loss of a chromosome resulted in a more favorable prognosis while a deletion was associated with a less favorable one fits with the finding that multiple structural chromosomal changes are associated with a poor prognosis while multiple numerical changes and few structural ones are associated with a good one [138]. This led He et al. [139] to use gene signatures for MYCN activity and chromosome deletions in 1p and 11q as well as complete loss of 11q to develop a computational algorithm from data obtained from studies of >2000 NB patients. Use of the algorithm to evaluate the data obtained for cells from a NB patient provided a significant improvement over other methods in predicting prognosis [139]. In another approach, droplet digital PCR was used to identify levels of seven different NB-associated mRNAs in bone marrow and blood. Results indicated that the values were significantly higher in patients that relapsed than in those who did not and provided a better prognosis than qPCR [140]. As our understanding improves about what the genetic modifications found in NBs mean in terms of proteins expressed and how they interact with other proteins and cell gangliosides it is anticipated that “personalized” medicine will be used to identify the most effective treatment modality for the patient.

## 7. Summary

From the above, it is evident that much progress has been made since the discovery of sphingolipids by Thudichum in 1884 [141]. In the case of the ganglio-series of sphingolipids, researchers have identified steps involved in their synthesis and continue to study the effects different ones have on cell behavior. Their effects on NB cell behavior have been compared to changes in ganglioside composition seen when neural stem cells differentiate into mature neural cells. The observation that just the oligosaccharide portion of GM1 could enhance process formation by NB cells [86] and reduce symptoms in a mouse model of Parkinson’s disease [108] underscores the need to define additional pathways affected when the oligosaccharide portion of a ganglioside interacts with molecules such as α-synuclein and galectins ([109,142] respectively).

As genetic approaches have advanced, research has concentrated on factors affecting translation of the genes encoding expression of proteins that can affect cell differentiation, proliferation, and adhesion. Interestingly, this led to the observation that, as discussed previously, hypermethylation of *B3GALT4* resulted in reduced synthesis of b-series gangliosides [48] while deacetylation of *ST3GAL5* and *ST8SIA1* enhanced expression of GD2 [124]. These observations provide a possible explanation for earlier findings that NB patients with tumors expressing higher concentrations of b-series gangliosides tended to have a better prognosis [19,20]. As genomic data have accumulated, it has become apparent that NBs display chromosome instability. Patients whose tumors were resistant to treatment were found to have high structural chromosomal variations while those whose tumors responded favorably exhibited numerical variations but few structural ones [138]. This raises the question of whether there are specific structural variations within the chromosomes that occur more frequently in tumors refractive to treatment and, if there are, what changes are induced in terms of translated products. Keeping in mind that modifications such as acetylation of the oligosaccharide portion of gangliosides can affect their interaction with proteins care must be taken to determine their presence/absence when interpreting effects. To optimize our understanding of why NBs behave as they do researchers need to integrate observations made about ganglioside function with information gained from genomic studies. As understanding of the functional effects of these changes improves, it should be possible to develop more effective therapeutics based on the individual characteristics of a patient’s tumor.

## Figures and Tables

**Table 1:** Classification of neuroblastomas.

INSS * (Uses Surgical Samples) [3]	INRGSS ** (Uses Imaging, Exams and Biopsies) [4]
Tumor confined to start site, no lymph node involvement	L1. Tumor confined to start site, no image-defined risk factors
2A. Tumor confined, no lymph node involvement, all cannot be removed surgically	L2. Tumor localized with one or more image-defined risk factors
2B. As for 2A but ipsilateral lymph nodes contain NB cells	
3. Tumor not removed entirely by surgery and cancer has crossed the midline; tumor is on one side but infected lymph nodes are contralateral; tumor is midline with bilateral lymph node involvement	
4. Metastatic disease	M. Metastatic disease
4S. As in stage 1 or 2 with metastases to liver, skin and/or bone marrow and child is <1 yr	MS. Tumor has metastasized to liver, skin and/or bone marrow and child is <18 months

* INSS: International Neuroblastoma Staging System; ** INRGSS: International Neuroblastoma Risk. Group Staging System.

**Table 2:** Examples of controls of activity of enzymes needed for synthesis of neural gangliosides from ceramide.

Transport Protein/Enzyme	Factor(s) Affecting Activity	Species *
CERT (ceramide transporter)	Phosphorylation by protein kinase D 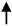 activity [26,27,28]	Human
Glc-cer synthase	Ceramide 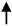 activity [36]	Human
FAPP2 (four–phosphate adaptor protein 2)	Failure to interact with acyl-coenzyme A binding domain 3 (ACBD3) [37]	Human
Lac-cer synthase (B4GalT5/6)	Sp1 transcription factor 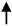 synthesis [38]	Human
GM3 synthase (ST3GAL5)	PKC 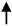 CREB-mediated transcription 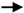 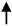 GM3 [39] 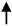 Specificity promotor 1 and activating protein 2 promote expression [40] Enzyme’s cytoplasmic tail determines activity, subcellular localization and stability [41]	Human Mouse Mouse
GD3 synthase (ST8SIA1)	N-glycosylation affects location and activity [42] NF-kB upregulates transcription [43]	Chicken Human
GA2/GM2/GD2 synthase (B4GALNT1)	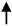 During neuronal differentiation [44] Coexistence of multiple isoforms [45] 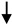 Sp1 or HDAC1 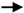 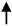 transcription [46]	Mouse Hamster Human
Sialidase 3 (NEU3)	BDNF 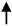 its activity [47]	Rat
GA1, GM1a, GD1b, and GT1c synthase (B3GalT4)	Gene hypermethylation 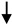 expression [48]	Human

* Animal sources are indicated as results may be species-dependent. Arrows pointing up indicate an increase, arrows pointing down, a decrease.

**Table 3:** Recent examples of cellular effects of gangliosides plus Glu- and Lac-cer.

Ganglioside	Effect	Cell Type
Glc-Cer	Anti-apoptotic, pro-survival 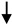 Endocytosis of transferrin receptor	Cancer cells [77] THP1 monocytes induced to become macrophage [78]
Lac-Cer	Lipid 2^nd^ messenger 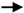 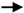 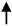 angiogenesis	Human endothelium [79]
GM3	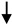 Lipopolysaccharide-induced inflammation by 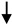 NF-κB, AP-1, and MAPKs signaling GM3 expression 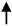 during oxidative stress due to 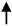 sialyltransferase activity	rAW 264.7 macrophage [80] Human neuroblastoma (NB) cells [81]
GM2	GM2, GM1, GD1a expression 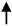 during oxidative stress due to 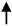 sialyltransferase activity	Human NB cells [81]
GM1	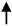 Movement of EGFR to caveolae 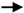 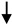 EGFR activity 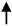 contact inhibition 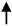 Insulin resistance in aging/senescence and inflammation 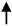 Dopamine and histamine post-synaptic binding 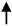 PI3K/AKT-Nrf2 pathway protects against high altitude-induced cerebral edema Oligosaccharide portion binds TrkA receptor 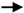 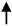 neurite outgrowth Tumor shed GM1 acts on macrophages 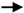 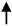 angiogenesis Reversed MK801 induced cognitive defects 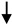 Autophagy following experimental stroke Binding by galectin 1 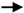 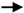 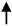 axon growth	Human mammary epithelial cells [82] Human endothelial cells [83] Model lipid bilayers [84] Rat brains [85] Murine NB cells [86] Macrophages [87] C57BL/6 J mice [88] Rats [89] Neurons and NB cells [90]
GD3	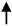 EGFR signaling to maintain cell self-renewal	Mouse neural stem cells [71]
GD2	Anti-GD2 antibodies induce nonclassical cell death Ab binding to GD2 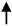 Src kinases 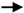 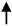 phosphorylation of NMDA receptor NR2B subunits, 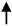 cAMP FAK-AKT-ERK-mTOR signaling 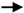 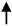 growth and invasion of cells 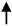 Angiogenesis	Tumor [91] NB cells [92] Breast cancer stem like cells [72] Melanoma and NB cells [93]
GD1a	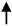 Expression during oxidative stress due to 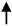 sialyltransferase activity	Human NB cells [81]
GT1b	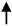 TLR2 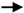 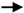 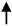 neuropathic pain	Spinal cord [94]

* For earlier information about the function of neuronal and neuroblastoma (NB) gangliosides see [95,96].

## Abbreviations

AKT	protein kinase B
ALK	anaplastic lymphoma kinase
CNS	central nervous system
EGFR	epidermal growth factor receptor
ERK	extracellular-signal-regulated kinase
FAC	focal adhesion kinase
FAPP2	4-phosphate adaptor protein 2
Fc	fragment crystallizable region of an antibody
Fv	fragment variable region of an antibody
GSL	glycosphingolipid
GM	monosialoganglioside
GD	disialoganglioside
GT	trisialoganglioside
HDAC	histone deacetylase
mTOR	mammalian target of rapamycin
MYCN	myelocytomatosis viral oncogene neuroblastoma derived homolog
NB	neuroblastoma
NEU	sialidase (neuraminidase)
NMDA	N-methyl-d-aspartic acid
NSC	neural stem cell
TLR2	toll like receptor 2
TrkA	tropomyosin receptor kinase A
